# Predicting lymph node metastasis of clinical T1 non-small cell lung cancer: a brief review of possible methodologies and controversies

**DOI:** 10.3389/fonc.2024.1422623

**Published:** 2024-12-09

**Authors:** Li Zhang, Feiyue Zhang, Gaofeng Li, Xudong Xiang, Haifeng Liang, Yan Zhang

**Affiliations:** ^1^ Department of Oncology, the Fifth Affiliated Hospital of Kunming Medical University, Gejiu, China; ^2^ Department of Thoracic Surgery, Yunnan Cancer Center, The Third Affiliated Hospital of Kunming Medical University, Kunming, China; ^3^ Department of Oncology, Yuxi City People’s Hospital, The Sixth Affiliated Hospital of Kunming Medical University, Yuxi, China

**Keywords:** clinical T1 non-small cell lung cancer, lymph node metastasis, lymph node dissection, noninvasive prediction, heterogeneity

## Abstract

Non-small cell lung cancer (NSCLC) is a major subtype of lung cancer and poses a serious threat to human health. Due to the advances in lung cancer screening, more and more clinical T1 NSCLC defined as a tumor with a maximum diameter of 3cm surrounded by lung tissue or visceral pleura have been detected and have achieved favorable treatment outcomes, greatly improving the prognosis of NSCLC patients. However, the preoperative lymph node staging and intraoperative lymph node dissection patterns of operable clinical T1 NSCLC are still subject to much disagreement, as well as the heterogeneity between primary tumors and metastatic lymph nodes poses a challenge in designing effective treatment strategies. This article comprehensively describes the clinical risk factors of clinical T1 NSCLC lymph node metastasis, and its invasive and non-invasive prediction, focusing on the genetic heterogeneity between the primary tumor and the metastatic lymph nodes, which is significant for a thoroughly understanding of the biological behavior of early-stage NSCLC.

## Introduction

Non-small cell lung cancer (NSCLC) is the most diagnosed subtype of lung cancer worldwide and poses a growing threat to human health (1). Despite the breakthroughs in precision molecular targeted therapies and immunotherapy have dramatically improved the treatment landscape for patients with advanced lung cancer (2, 3), significantly reducing mortality in lung cancer patients is attributed to the low-dose computed tomography (LDCT)-based lung cancer screening in high-risk populations, which has resulted in an increasing number of early-stage lung cancers being detected especially clinical T1 NSCLC (tumor size ≤ 3 cm) (4). Furthermore, early cancer detection creates time window to minimize lymph node metastasis(LNM) (5). LNM is the most common metastatic route and the most critical factor affecting the prognosis of NSCLC. However, there is still no consensus on the lymph node detection and dissection strategy for clinical T1 NSCLC (6, 7). In addition, accurate identification of lymph nodes and prediction of tumor spread risk, as well as tumor mutational heterogeneity, are critical for guiding optimal staging and personalized treatment of NSCLC patients (8).

## Clinical factors of lymph node metastasis of clinical T1 NSCLC

**Figure 1:**
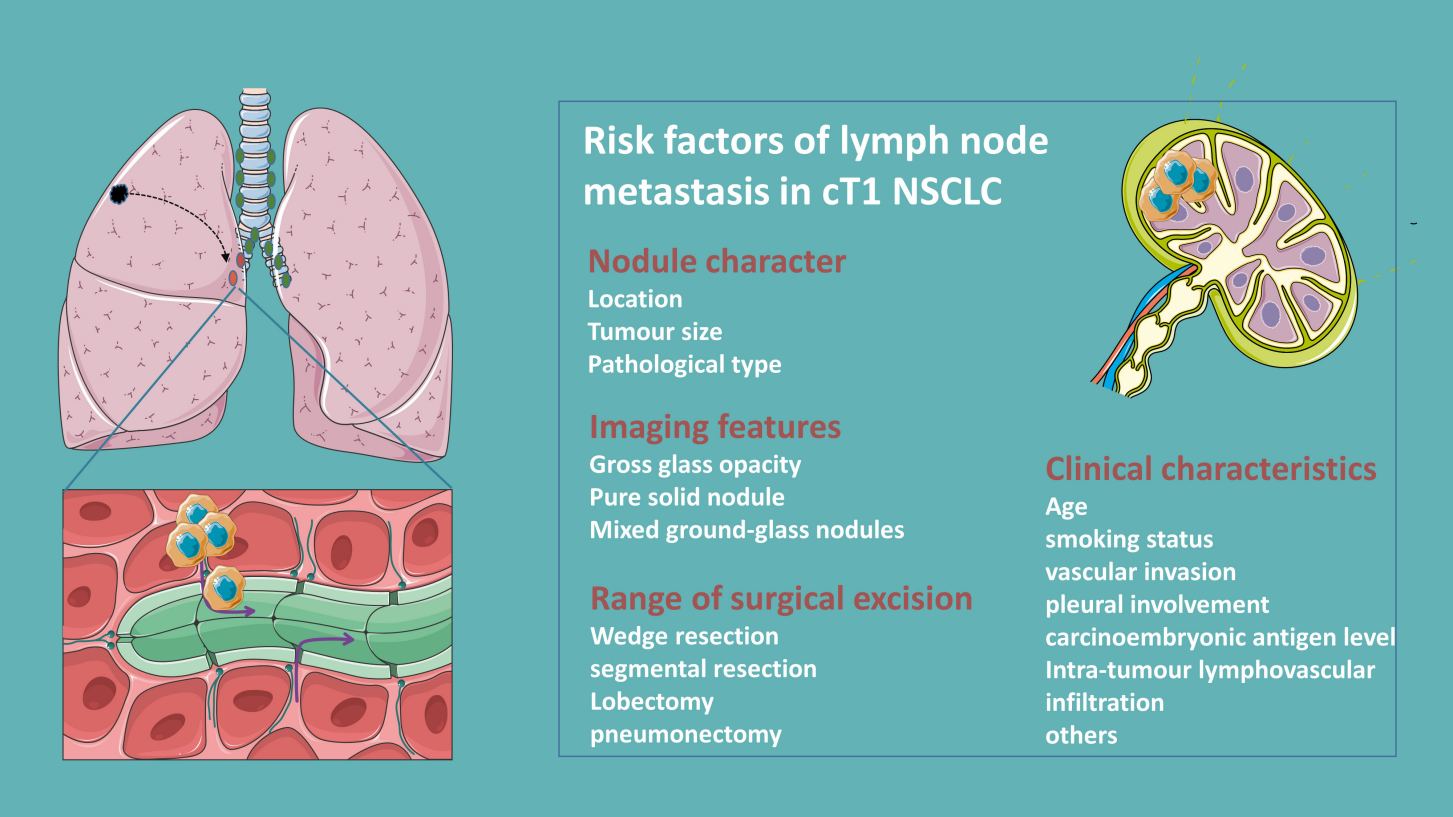
Clinical risk factors of lymph node metastasis in clinical T1N0 NSCLC.

LNM in NSCLC patients is strongly associated with age, smoking status, tumor size, histology and differentiation, carcinoembryonic antigen level, and vascular invasion (+) and pleural involvement (+) (9–11). The pattern of regional LNM in clinical T1 peripheral NSCLC is significantly influenced by tumor size (12). Compared to primary tumor size, a large solid portion is a more critical predictor of LNM in patients with clinical T1 partially solid lung adenocarcinoma (13, 14). Moreover, patients with micropapillary or solid components are associated with LNM, while the gross glass opacity (GGO) components or microscopic invasive adenocarcinomas ≤2.0 cm or invasive mucinous adenocarcinomas are less prone to LNM (15–17). Besides, centrally located T1 tumors also predict a higher risk of pathological upstaging (18, 19) **Figure 1**.

## Lymph node dissection strategies for clinical T1N0 NSCLC

Lymph node dissection (LND) is categorized into systemic/lobe-specific mediastinal lymph node dissection (m-LND) and hilar lymph node dissection (h-LND) only according to its extent (20). Selective LND is feasible in patients with clinical T1N0 NSCLC with predominantly GGO tumors, and pure GGO subproportion even do not need LND given the approximate 0% rate of LNM (21, 22). Interestingly, some studies show there is no significant correlation between LNM rate and tumor size for patients in the ≤2 cm mixed ground glass nodule (mGGN) group, and lobe-specific m-LND was suggested for patients with a solid component ≤2 cm pure solid nodule (PSN) (23–25). Furthermore, different primary tumor lung lobe locations with different propensities for mediastinal lymph node metastasis are observed in clinical T1N0 peripheral NSCLC just as for tumors in the upper lobe (≤3 cm), lower m-LND is not required, whereas for tumors in the lower lobe (≤2 cm), upper m-LND is not necessary (26, 27). However, occult nodal disease in patients with small (≤2 cm) nodes of clinical N0 NSCLC often occurs at the peripheral N1 station (11-13), therefore h-LND is essential for accurate staging in patients with clinical N0 NSCLC (28). Yet the controversy remains, a multicentric evaluation study reported that pN1 status in cN0 patients with central NSCLC tumors was observed in up to 27% of cases (29). This is inconsistent with newer studies reporting occult N1 rates of only between 2-3% (30, 31). Differently, some demonstrate that routine dissection of the aortopulmonary zone and inferior mediastinal nodes is sufficient to ensure staging accuracy, and more LND does not improve survival but may increase the risk of postoperative complications (32). Clinical parameters such as lung membrane invasion, vascular invasion, and carcinoembryonic antigen (CEA) were applied to stratify the risk of LNM, which in turn led to different LNDs (33). Others, such as intratumoral lymphovascular infiltration, are risk factors for LNM in patients with NSCLC, and adjuvant therapy should be considered for such patients (34).

Due to tumor trans-airspace spread, sublobar resection is associated with regionally occult lymph node metastasis and further stratifying patients with stage IA lung adenocarcinoma on the risk of recurrence according to the extent of resection (wedge resection > segmental resection > lobectomy) (35). Additionally, video-assisted thoracoscopic surgery and robotic lobectomy have a lower rate of pathologic LN upstaging after lobectomy compared with conventional open-thoracic surgery (36). Surgeons should thoroughly evaluate hilar and mediastinal nodal metastases and select a reasonable LND.

## Noninvasive quantitative prediction of lymph node metastasis in clinical T1N0 NSCLC

Radiologists’ assessment of lymph node status based on preoperative CT lacks high accuracy for patients with early-stage lung cancer and is inefficient. Combining CT radiological features with clinical histopathological models of the primary tumor and lymph nodes shows great potential in predicting lymph node metastasis in resectable NSCLC. More importantly, the pre-surgical CT-based radiomics model performed better than the clinical model in predicting LNM in stage IA NSCLC patients, and can be used for non-invasive quantitative prediction of mediastinal LNM in lung adenocarcinoma (37, 38). Using inner margin ratio (IMR) and outer margin ratio (OMR) thresholds are capable of predicting N1 metastases in patients with clinical T1 NSCLC staged on imaging (39), and electron density (ED) derived from dual-energy CT (DECT) is useful in the diagnosis of LNM in NSCLC (40). Some textural features from CT are associated with the degree of malignancy of mediastinal lymph nodes (41). Models of radiomic features extracted from gross tumor volume (GTV), peritumor volume (PTV), and CT histogram analysis of tumors could be used for preoperative prediction of LNM in T1 peripheral lung adenocarcinoma. Besides, CT-based radiological consensus clustering is able to identify associations between radiological features and clinicopathological and genomic features and prognosis (42–45).

Deep machine learning of radiologists’ CT readings and their clinical information can be used to guide clinical management of high-risk populations following screening CT (46). Swin Transformer-based deep learning features in predicting LNM outperforms radiomics features and clinical semantic models in extracting common multilevel features from high-resolution 3D CT images, where the Feature Dynamic Transfer (FDT) module facilitates the ability to recognize LNM (47, 48). Additionally, a cross-modal 3D neural network deep learning approach based on CT images and prior clinical knowledge performed significantly better than the radiomics approach and radiologists, improving the diagnostic accuracy of predicting LNM in clinical stage T1 lung adenocarcinoma (49, 50).

PET/CT is more accurate and sensitive than CT for mediastinal staging (51). Lymph nodes in NSCLC patients are usually evaluated using [18F]FDG-PET/CT, and DTP PET/CT using a semiquantitative technique has demonstrated good diagnostic performance in detecting mediastinal LNM in NSCLC patients (52, 53). Metastatic lymph nodes showed higher FDG uptake than false-positive lymph nodes (54). Lung invasive adenocarcinomas with micropapillary or solid components had higher SUVmax, MTV, TLG, and were associated with LNM. SUVmax had high specificity in predicting mediastinal LNM in 10.1-30.0 mm solid NSCLC. Assessment of SUVmax on a 5-point scale using the Deauville score is helpful in predicting LNM in early-stage lung adenocarcinoma. Lymph nodes SUVmax is also associated with the presence of tumor-promoting stromal cells in LNM (55–58). TLGsur showed strong predictive performance in predicting occult lymph node metastasis in clinically node-negative (cN0) lung adenocarcinoma (59). However, FDG PET/CT has a limited role in the preoperative detection of lymph nodes or distant metastases in patients with sub-solid NSCLC with solid portions 3 cm or smaller in size (60).

A machine learning model based on [18F]FDG-PET/CT routinely available variables improves the accuracy of mediastinal LN staging compared to established visual assessment criteria; a support vector machine (SVM) model is able to predict metastatic lymph nodes, and a machine-learning-based model-free algorithm for generating probabilistic maps based on a number of spatial and temporal features of 18F-FDG uptake can improve the specificity of distinguishing adenocarcinoma and its identification of metastatic lymph nodes (61–63). Machine learning models that incorporate clinical information into quantitative variables of 18F-FDG PET/CT can improve the diagnostic accuracy of LNM. The Tumor and Lymph PET/CT Clinical Model (TLPC) model can noninvasively predict LNM in NSCLC, which can help clinicians develop more rational treatment strategies (64, 65).

Circulating tumor DNA (ctDNA) has emerged as a non-invasive biomarker for dynamic tumor monitoring and is a non-invasive means of assessing intra-tumor heterogeneity (66). For instance, high Variant allele frequency (VAF) levels in preoperative ctDNA may predict LNM in resectable NSCLC (67). Compared with conventional radiography, ctDNA analysis can detect the smallest residual lesions of resectable NSCLC, thus facilitating early intervention (68). Utilizing ctDNA-based liquid biopsies with help to gain insight into the process of metastatic spread (69). A ctDNA-based preoperative noninvasive prediction model for LNM in patients with resectable NSCLC has satisfactory discrimination and calibration.

One-step nucleic acid amplification (OSNA) is a rapid intraoperative molecular testing technique to quantitatively assess tumor burden in resected lymph nodes of lung cancer patients by quantitatively measuring keratin 19 (CK19) mRNA, which provides high diagnostic accuracy and speed for detection of LNM, and can be applied to intraoperative decision-making for personalized lung cancer surgery (70, 71). Moreover, folate receptor-positive circulating tumor cells have a predictive value for the preoperative diagnosis of LNM (72). More and more measures are developed, as a zebrafish tumor xenograft zebrafish tumor xenograft model based on implantation of Patient-derived xenograft tissue fragments has high sensitivity for predicting LNM (73).

## Invasive staging of lymph nodes

In NSCLC patients at risk for LNM, invasive mediastinal lymph node staging prior to curative resection is associated with significantly improved survival (74). EBUS-TBNA has been established as a first-line staging option for patients with lung cancer (75). Especially in patients with central clinically staged T1N0M0 NSCLC, EBUS-TBNA provide an extraordinary diagnostic accuracy for mediastinal staging (76). Therefor, guidelines recommend invasive mediastinal staging for patients with centrally locate NSCLC, however, the selection of candidates for invasive mediastinal staging for patients with clinical T1N0M0 lung cancer based solely on the location of the central tumor is controversial (77). Some studies demonstrate that subsequent invasive staging may not be necessary for those with peripheral T1 tumors with a prominent ground-glass component after a negative PET-CT (18).

Additionally, micrometastases such as occult lymph node metastasis(OLM) should be selected for mediastinoscopy or EBUS-TBNA (78). Accurate pN0 diagnosis depends on the number of LNs examined, which in turn quantifies the risk of OLM in patients with pN0 NSCLC, and a risk stratification model categorizes EBUS-TBNA-negative lymph nodes into different risk groups (79, 80). Metastatic hilar or mediastinal lymph nodes can be effectively obtained by EBUS-TBNA or the convex probe EBUS (CP-EBUS), and further EGFR, KRAS evaluation is not inferior to conventional lung cancer tissue samples (81, 82).

Unlike EBUS-TBNA bronchoscopy which is used primarily via the airway, endoscopic ultrasound with bronchoscopy-guided fine-needle aspiration (EUS-B-FNA) is used transesophagically for evaluation of lesions that cannot be accessed via the airway are gradually attracting the attention of oncologists. EUS-B-FNA improves the diagnostic yield of EBUS bronchoscopy for intrathoracic lesions (83). EUS-B-FNA is also a safe and accurate method for the diagnosis of paraesophageal lung lesions (84). Furthermore, the combination of EBUS and EUS can significantly improve the sensitivity of detection of mediastinal nodal metastases, thereby reducing the need for surgical staging (85). However, given the low incidence of occult mediastinal metastases and the poor sensitivity of endoscopy in this population, strategies for invasive mediastinal staging need to be adapted accordingly (86).

## Mutational heterogeneity of primary tumor and metastatic lymph nodes in NSCLC

In addition to clinical and/or pathological diagnosis, comparison of driver mutation profiles of primary lung cancer tumors and their LNM can further differentiate between primary and metastatic tumors. Intratumor heterogeneity is the presence of multiple genetically distinct populations within a primary tumor, providing the basis for tumor metastasis (87). There are differences in the mutation profiles of key genes such as EGFR between primary lesions and metastatic lymph nodes in NSCLC (88, 89). Numerous studies have shown that lymph node metastatic status is associated with mutations in NSCLC driver genes (90). Different genotypes of NSCLC have different propensities for LNM, cases with fusion mutations have a higher risk and burden of LNM than other genotypes, and EGFR mutations are associated with N2 jump metastasis (N2 lymph node metastasis in the absence of N1) in lung adenocarcinoma (91–94). Increasingly, novel genes such as SMARCA1, SMARCA4 and SMAD4 alterations in lung adenocarcinoma are independently associated with LNM in lung adenocarcinoma (95, 96). The EGFR mutation status of metastatic lymph nodes also serves as a predictor of response to EGFR-TKI therapy in patients with recurrent NSCLC after surgical resection (97). Consequently, differences in gene mutation status between the primary tumor and the corresponding LNM should be taken into account when formulating a tyrosine kinase inhibitor-targeted treatment regimen (98).

In contrast, some researchers illustrate that given the minimal functional driver gene heterogeneity in primary-metastasis, a single biopsy of the primary tumor is sufficient to capture the majority of functionally important mutations in metastases (99, 100). Contradictory findings may be related to the means of detection, with ALK results detected by FISH showing more frequent inconsistencies between primary tumors and matched metastases compared to IHC, which may be due to the technique and the quality of the samples (101). Therefore, laboratory quality control of samples and the corresponding technical standards for testing should be continuously optimized.

## Discussion

More and more clinical T1N0 NSCLC are detected and effectively intervened with good prognosis, and the existence of heterogeneity between NSCLC primary tumor and metastatic lymph node lesions has been increasingly revealed with the application of high-throughput sequencing technology. However, the LNM pattern and the specific mechanism of early-stage lung cancer still need further research. Given early-stage lung cancer patients with high risk of LNM should be subjected to more intensive surveillance strategies after radical surgical treatment, developing the risk-predicting mold combining clinical and tumor genomic features which are capable of identifying patients at risk of pathological LNM is warranted. Especially, new approaches such as the use of validated predictive models that combine radiomics and ctDNA for noninvasive prediction will help to better select patients, still needs to be confirmed by prospective studies. Consequently, optimal preoperative lymph node assessment and prediction, as well as the extent of intraoperative lymph node dissection need to be confirmed by large-scale clinical randomized controlled trials.
